# Human Papilloma Virus (HPV) Oral Prevalence in Scotland (HOPSCOTCH): A Feasibility Study in Dental Settings

**DOI:** 10.1371/journal.pone.0165847

**Published:** 2016-11-18

**Authors:** David I. Conway, Chris Robertson, Heather Gray, Linda Young, Lisa M. McDaid, Andrew J. Winter, Christine Campbell, Jiafeng Pan, Kimberley Kavanagh, Sharon Kean, Ramya Bhatia, Heather Cubie, Jan E. Clarkson, Jeremy Bagg, Kevin G. Pollock, Kate Cuschieri

**Affiliations:** 1 School of Medicine, Dentistry and Nursing, University of Glasgow, Glasgow, United Kingdom; 2 Mathematics and Statistics, University of Strathclyde, Glasgow, United Kingdom; 3 Glasgow Caledonian University, Glasgow, United Kingdom; 4 NHS Education for Scotland, Dundee, United Kingdom; 5 MRC/CSO Social and Public Health Sciences Unit, University of Glasgow, Glasgow, United Kingdom; 6 Sandyford Sexual Health Services, NHS Greater Glasgow and Clyde Health Board, Glasgow, United Kingdom; 7 Centre for Population Health Sciences, Usher Institute, University of Edinburgh, Edinburgh, United Kingdom; 8 Robertson Centre for Biostatistics, University of Glasgow, Glasgow, United Kingdom; 9 Human Papillomavirus Research Group, Division of Pathology, University of Edinburgh, Edinburgh, United Kingdom; 10 School of Dentistry, University of Dundee, Dundee, United Kingdom; 11 Health Protection Scotland, National Services Scotland, Glasgow, United Kingdom; 12 Scottish HPV Reference Laboratory, Royal Infirmary of Edinburgh, Edinburgh, United Kingdom; Fondazione IRCCS Istituto Nazionale dei Tumori, ITALY

## Abstract

The purpose of this study was to test the feasibility of undertaking a full population investigation into the prevalence, incidence, and persistence of oral Human Papilloma Virus (HPV) in Scotland via dental settings. Male and female patients aged 16–69 years were recruited by Research Nurses in 3 primary care and dental outreach teaching centres and 2 General Dental Practices (GDPs), and by Dental Care Teams in 2 further GDPs. Participants completed a questionnaire (via an online tablet computer or paper) with socioeconomic, lifestyle, and sexual history items; and were followed up at 6-months for further questionnaire through appointment or post/online. Saline oral gargle/rinse samples, collected at baseline and follow-up, were subject to molecular HPV genotyping centrally. 1213 dental patients were approached and 402 individuals consented (participation rate 33.1%). 390 completed the baseline questionnaire and 380 provided a baseline oral specimen. Follow-up rate was 61.6% at 6 months. While recruitment was no different in Research Nurse vs Dental Care Team models the Nurse model ensured more rapid recruitment. There were relatively few missing responses in the questionnaire and high levels of disclosure of risk behaviours (99% answered some of the sexual history questions). Data linkage of participant data to routine health records including HPV vaccination data was successful with 99.1% matching. Oral rinse/gargle sample collection and subsequent HPV testing was feasible. Preliminary analyses found over 95% of samples to be valid for molecular HPV detection prevalence of oral HPV infection of 5.5% (95%CI 3.7, 8.3). It is feasible to recruit and follow-up dental patients largely representative / reflective of the wider population, suggesting it would be possible to undertake a study to investigate the prevalence, incidence, and determinants of oral HPV infection in dental settings.

## Introduction

Oropharyngeal (throat) cancer (OPC) is among the cancers with the fastest increasing incidence in the UK, with a near 3-fold rise in the past 15 years and incidence rates around 5 per 100,000 [1, 2, 3]. These trends are a global phenomenon—described as an epidemic in North America [4], with rapid increases also observed in Central Europe and South America following behind this curve [5]. Around 60% of OPC is associated with oral Human Papillomavirus (HPV) infection; likely acquired from genital HPV infection during oral sexual behaviour, with smoking also a strongly associated risk factor [6, 7].

HPV infection is increasingly implicated in the rising incidence of OPC [8], with HPV types 16/18 responsible for almost all HPV-positive cases of OPC [9]. The natural history of oral HPV infection—including prevalence, incidence, determinants and risk factors—is relatively unknown. To date, the only large population-based study of oral HPV prevalence has been undertaken in the United States of America [10]. This cross-sectional study (n = 5579) found an overall population prevalence of 6.9% and a bimodal increasing pattern with age—a prevalence of over 7% among those in their 20s and 30s, and a further peak of over 11% in those in their 50s and early 60s. Another cohort (n = 1688) of men from across the Americas found an overall prevalence of 4% [11] and 4.4% incident oral HPV infections in 12 months [12], and a small cohort (n = 212) of male university students in the USA reported a prevalence of 7.5% [13]. An earlier systematic review of 18 cancer case-control studies worldwide found an overall prevalence of 4.5% oral HPV infection in healthy individuals (cancer-free control subjects) [14].

There have been only two European studies, both small. In the first, a 9.3% prevalence was found in a Swedish sexual health clinic-based study (n = 483), of adults aged 15–23 years, with reported high cervical HPV prevalence (70%) among the female participants (n = 408) [15]. In the second, a 1.2% prevalence was reported in a small sample (n = 81) study of hospital outpatients in Italy, aged 49–77 years [16].

Oral HPV prevalence or incidence have rarely been investigated in relation to HPV vaccination. One proof-of-principle study demonstrated the prevention potential of the HPV vaccination against oral HPV infection [17]. A recent follow-up study of the aforementioned Swedish study found a substantial decrease in oral HPV prevalence to 1.4% in 2013–14 from 9.3% in 2009–11 following the introduction of HPV vaccination between 2007 and 2014 [18].

The National HPV Immunisation Programme in Scotland began in 12–13 year old females in 2008 as an intervention to reduce cervical cancer. Uptake of the HPV vaccine has been impressive, with sustained high levels of coverage (>90%) and a catch-up campaign, running over 3 years, which offered vaccination to all females aged 13–18 years old with an uptake of 66% [19]. It is feasible that the immunisation programmes will have an impact on infection and disease beyond those recently demonstrated for the anogenital tract [20]. However, there are no routine data on the population-based incidence and prevalence of oral HPV, nor on the impact of immunisation on oral infection or herd immunity in males.

The primary aim of the HOPSCOTCH project was to test the feasibility of using dental settings, to undertake a population-wide (16–69 years) epidemiological study of the incidence, prevalence, and determinants of oral HPV infection in the Scottish population, which could ultimately allow assessment of the impact of the HPV vaccination programme in Scotland on oral HPV infection in young women and potential herd immunity in men aged 16–25 years old. The specific objectives of the study were to assess the feasibility and best methods of recruiting dental patients and capturing complete questionnaire data (including sexual history); and to examine the practical / logistical aspects of taking and transferring oral gargle/rinse samples from the dental facility to the Scottish HPV Reference Laboratory in a state suitable for assay. NHS dental facilities (including dental practices and dental outreach teaching centres) in Scotland are potentially ideal settings for this study as patients are generally healthy, attend regularly for routine dental appointments [21], and the oral gargle/rinse method of specimen collection is in-keeping with dental clinical activities. Dental teams also have a potential role in tackling rising oral/oropharyngeal cancer trends through prevention and early detection [22]. Moreover, Scotland is particularly well placed to conduct this research given the robust national health datasets and data linkage potential at the individual patient level, allowing individual vaccination histories to be obtained [19, 23].

## Methods

### Recruitment

Baseline recruitment commenced in November 2013 and continued to March 2015. Potential outreach teaching centres and General Dental Practitioners (GDPs) registered with the Scottish Dental Practice Based Research Network (SDPBRN) as Rapid Evaluation Practices (REPs) were sent a Study Information Sheet and invitation letter and subsequently contacted by telephone to confirm suitability for participation. All potential sites were assessed for suitability regarding their geographical location, patient numbers/profile, wi-fi/internet accessibility and availability of private space. Baseline patient recruitment and data collection took place in seven sites (three outreach teaching centres and four GDPs). We tested recruitment and data collection conducted by Research Nurses in five sites (initially two, but extended to three outreach teaching centres and two GDPs) versus recruitment and data collection conducted entirely by the dental practice clinical team (in two further practices). Sites were based in NHS Greater Glasgow & Clyde (GG&C) and NHS Tayside Health Boards (S1 File). Relevant personnel, including Research Nurses and Dental Care Teams from all participating sites, were provided with study-specific training, including how to handle queries and concerns raised by the questionnaire topics. Dental practice participation costs for each of the two models were developed based on similar items of service on the NHS Scotland primary care dental contract and were set in agreement with participating dentists as a reasonable fee for the tasks (S2 File).

A paper-based participation/screening log was used to record the details of all patients screened for eligibility. Screened patients were either: i) sent the study invitation materials (invitation letter, Participant Information Sheet—which included the information that we would not be providing participants with the results of the HPV test) two weeks prior to routine dental appointment; or ii) given the materials at the time of appointment, provided this was at least 30 minutes ahead of the approach for potential recruitment to the study. The method of approach varied by site, related to local patient appointment processes. Participating patients were assigned a study identification number and contact information was stored for the 6-month follow-up. For non-participants, reasons for non-participation, age, sex and home postcode (to assign the area-based socioeconomic deprivation index score—Scottish Index Multiple Deprivation (SIMD) [24])–were logged.

Informed consent was sought by Research Nurses at five sites, and by a trained Dental Nurse from the Dental Care Team at two sites. Consent was sought for study participation (including 6-month follow-up), specimen storage in the Scottish HPV Archive (Edinburgh), participation in data linkage, and willingness to be approached to participate in future studies.

We developed a bespoke, web-based questionnaire with the University of Glasgow Robertson Centre for Biostatistics (RCB). Responses were stored directly onto the RCB secure servers. Participants self-completed the questionnaire via an online tablet computer in a private area (paper versions were also available where needed—S3 File—female questionnaire and S4 File—male questionnaire). Items were based on previous studies in the field [10], along with the National Survey of Sexual Attitudes and Lifestyles (NATSAL) [25] and the Scottish Health Survey [26]. These included demographics (age, gender, ethnicity, socioeconomic status), lifestyle behaviours (use of tobacco, alcohol and other recreational drugs), oral health and dental care, sexual behaviour history / health, and history of HPV vaccination.

### Follow-up at 6-months

Participants were invited to provide follow-up data 6-months after baseline in one of the three following ways:

1. Participants recruited via the Research Nurse model were followed up via specific study appointment sessions. All follow-up activities were conducted by a Research Nurse. Participants were reimbursed for travel costs.
2. Participants recruited via the GDP model were followed up via return to the general dental practice setting at routine 6 monthly dental recall appointments. Follow-up activities were conducted by the Dental Care Team.
3. The final one-third (sequentially) of all participants (both recruitment models) was followed up via a postal/online questionnaire, with self-administration of the oral gargle/rinse and posting of the sample. Those who failed to attend their follow-up appointment were also offered this option.

### Data-linkage

The study examined the feasibility of successfully linking patient participant data to routine health datasets. A unique identifier relating to each participant who consented to record linkage to national datasets, including the Scottish Immunisation & Recall System (SIRS) and Child Health Schools Programme-System (CHSP-S) for HPV vaccination data, was assigned to both his/her consent form and questionnaire. Data linkage was performed by the Electronic Data Research and Innovation Service (eDRIS), Information Services Division, NHS National Services Scotland.

### Oral gargle/rinse specimen collection

Participants provided a bio specimen comprising a 10 ml oral rinse sample in saline mouth rinse at two time points, 6 months apart [10, 25]. Participants performed the oral gargle/rinse for 30 seconds in total—timed on a timer application on the tablet computer. Specimens were sealed, labelled with the participant identifier and stored at room temperature or in a fridge until they were transported to the Scottish HPV Reference Laboratory (SHPVRL) for processing, nucleic acid (NA) extraction and molecular HPV testing.

### Specimen processing / testing

Cell pellets were obtained from 10 ml rinses via centrifugation at 2900 ± 150 X g for 15 ± 2 mins., supernatants were discarded and the cellular pellets frozen in tissue stabilisation buffer (Qiagen, Manchester UK) pending NA extraction. NA was extracted using a system initially optimised for downstream HPV detection of cervical and urine samples [13] using the Qiagen Media Kit (Qiagen, Manchester, UK) on the MDX Platform. Yield and quality of NA were measured by spectrophotometry (260/280 ratios). Residual extracts after HPV genotyping were stored in the Scottish HPV Archive (http://www.shine.mvm.ed.ac.uk/archive.shtml) for use in anonymised research in the future.

### HPV genotyping

HPV amplification and genotyping were performed using a luminex based HPV genotyping assay, currently used for national HPV immunisation surveillance [27, 28]. This assay is based on amplification of a short fragment of the HPV L1 region, and delineates up to 24 high and low risk HPV types (including those types covered by the vaccine). It also incorporates a beta globin control for cellularity. HPV type specific prevalence was analysed descriptively.

Statistical analysis of participant data was carried out using R version 3.1.1. Confidence intervals for the participation proportions, follow-up rates and HPV positivity percentages were calculated using Wilson’s method using the Hmisc package in R. 95% confidence intervals are reported in all cases. Chi square tests were used to test for associations between the outcomes and demographic variables. Simulation tests based upon 5000 replications were used to calculate the p values. Post stratification weights were estimated using raking to adjust the HPV prevalence to address any imbalance between sample and population demographics. This was carried out using the survey package in R.

### Ethics statement

NHS West of Scotland Research Ethics Committee (REC) specifically approved this study [Reference no. 13/WS/0166:19/09/13], and NHS National Services Scotland Privacy Advisory Committee (PAC) [PAC37/14:4/02/15] approved the data linkage.

## Results

### Participation rates

A total of 1212 individuals, aged 16–69, were approached to take part in the study from seven centres in two health board areas—NHS GG&C and NHS Tayside. Of these, 59% were female compared to the Scottish population of which 51% in this age-group are female [29]. The age distribution among the individuals who were approached was also an under-representation of those in the 16–25 age group (12% of those approached and 19% of population) and the 26–39 age group (20% of those approached and 25% of the population). There is a corresponding slight over-representation in the older ages (Table 1).

**Table 1:** Participation rates by demographic and research model characteristics.

Variable	Category	Approached n	Participated n	%	95% CI	
**Overall**		1212	402	33.2	30.6	35.9
**Gender**	**Male**	502	161	32.1	28.1	36.3
	**Female**	710	241	33.9	30.6	37.5
**Age**	**16–25**	146	42	28.8	22.0	36.6
	**26–39**	245	106	43.3	37.2	49.5
	**40–49**	255	84	32.9	27.5	38.9
	**50–59**	243	90	37.0	31.2	43.3
	**60–69**	183	80	43.7	36.7	51.0
	**Missing**	140	0	0.0	0.0	2.7
**SIMD**	**1 (most deprived)**	280	90	32.1	26.9	37.8
	**2**	157	59	37.6	30.4	45.4
	**3**	177	61	34.5	27.9	41.7
	**4**	279	92	33.0	27.7	38.7
	**5 (least deprived)**	265	90	34.0	28.5	39.9
	**Incomplete Postcode**	40	6	15.0	7.1	29.1
	**Missing Postcode**	14	4	28.6	11.7	54.6
**Centre**	**Teaching/outreach 1—Tayside**	102	17	16.7	10.7	25.1
	**GDP 1—Tayside**	176	34	19.3	14.2	25.8
	**GDP 2—Tayside**	268	104	38.8	33.2	44.8
	**Teaching/outreach 1—GG&C**	369	130	35.2	30.5	40.2
	**GDP 1—GG&C**	167	60	35.9	29.0	43.4
	**GDP 2—GG&C**	101	45	44.6	35.2	54.3
	**Teaching/outreach 2—Tayside**	29	12	41.4	25.5	59.3
**Recruitment model**	**Research Nurse**	935	323	34.5	31.6	37.7
	**Dental Team**	277	79	28.5	23.5	34.1
**Setting**	**Teaching/outreach**	500	159	31.8	27.9	36.0
	**General Dental Practice (GDP)**	712	243	34.1	30.7	37.7

n—number; CI—Confidence Interval; SIMD—Scottish Index of Multiple Deprivation; GDP—General Dental Practice; GG&C—Greater Glasgow & Clyde

In total, 402 individuals agreed to participate in the study giving an overall participation rate of 33.1% with no imbalance in participation rates between men and women. Among the 1072 with known age, participation was significantly associated with age (p = 0.010) and was lower among those aged 16–25. There was no apparent association (p = 0.827) with participation and SIMD (Table 1).

There were significant differences among the participation rates according to research site—(p<0.0001), with relatively low participation in two centres (GDP1 –Tayside, and outreach teaching 1 –Tayside). Participation was not significantly (p = 0.072) associated with the use of a Research Nurse as opposed to a Dental Care Team. Similarly, it was not associated (p = 0.432) with the type of dental setting (Table 1). Reasons for non-participation were documented for 81% of the 811 non-responders (Table 2). The main reason noted for non-participation was patient refusal (31.6%).

**Table 2:** Reasons for non-participation.

Reason for Non Participation	n	%
Did not want to participate	256	31.6
Did not attend appointment	132	16.3
Did not have time	107	13.2
Did not receive Patient Information Sheet	43	5.3
Dentist refused	7	0.9
Inability to read English	5	0.6
Did not want to complete questionnaire	4	0.5
Concerned about NOT receiving HPV test results	2	0.2
Did not want to complete oral rinse/gargle	2	0.2
Did not want to commit to 6-month follow-up	1	0.1
Other	96	11.8
Not Known	156	19.2
Total	811	

Of those who had agreed to participate (n = 402): (a) all participants had initialled the box on the consent form indicating they had understood the Participant Information Sheet and agreed to participate in the baseline and 6-month follow-up; (b) 16 (3.9%) did not give consent for the data linkage element of the study; (c) 23 (5.7%) participants did not wish to be contacted about future studies. The Research Nurse model was more effective in terms of achieving the target recruitment numbers on time in three of the four sites (26 recruiting weeks, 30 calendar weeks), while the Dental Care Team model did not reach the target despite recruiting in almost twice the number of recruiting weeks (49 recruiting weeks, 55 calendar weeks). The average cost per recruitment was substantially lower in the Dental Care Team model (£26.23)–under half those associated with the Research Nurse (£66.46).

### Questionnaire data

A total of 390 participants (from 402 who consented) completed the questionnaire (n = 210, 53.8% electronically, and n = 180, 46.2% on a paper version); 263 (67.4%) participants completed all 30 questions which should have been answered by everyone, 354 (90.8%) omitted less than 2 questions. The most non-responses (12.1%) were for the income question (Table 3). Only 7 (1.8%) participants did not answer the question about recreational drug use (Table 4). Similarly there were low levels of missing data on the sexual health questions for both men and women and there was only one woman who did not answer the HPV vaccination question. Most participants (99.0%) answered some of the sexual history questions, and 331–136 men / 195 women–(84.9%) answered all seven of the questions for men and all eight for women (Table 5). While we had high responses to the sexual behaviour and health questions, 35.7% of males and 39.6% of females reported that they were uncomfortable with the questions on sexual activity in feedback items at the end of the questionnaire.

**Table 3:** Participant self-report sociodemographic characteristics.

Characteristic		Number Responding	Male % (n = 158)	Female % (n = 232)
**Ethnicity**	**White**	383	93.5% (143)	95.7% (220)
	**Mixed**		0.7% (1)	0.0% (0)
	**Asian; Asian Scottish or Asian British**		2.6% (4)	2.2% (5)
	**African**		1.3% (2)	1.3% (3)
	**Any other ethnicity**		2.0% (3)	0.9% (2)
	**Missing n**		5	2
**Education level**	**Primary School**	377	0.0% (0)	0.9% (2)
	**Secondary School**		15.5% (23)	21.8% (50)
	**Further Education/Technical College**		39.2% (58)	29.7% (68)
	**University**		40.5% (60)	40.6% (93)
	**Some other type of college**		4.7% (7)	7.0% (16)
	**Missing n**		10	3
**Marital status**	**Never married and never registered a same-sex civil partnership**	370	28.9% (43)	34.8% (77)
	**Married**		58.4% (87)	40.3% (89)
	**Separated but still legally married**		2.0% (3)	5.4% (12)
	**Divorced**		8.1% (12)	12.2% (27)
	**Widowed**		1.3% (2)	4.5% (10)
	**In a registered same-sex civil partnership**		1.3% (2)	2.7% (6)
	**Missing n**		9	11
**Annual income (£)**	**Nil or loss**	343	1.4% (2)	2.0% (4)
	**1-9,999**		10.5% (15)	18.5% (37)
	**10k-19,999**		20.3% (29)	26.5% (53)
	**20k-29,999**		14.7% (21)	16.0% (32)
	**30k-39,999**		19.6% (28)	13.5% (27)
	**>40k**		33.6% (48)	23.5% (47)
	**Missing n**		15	32

**Table 4:** Participant self-report lifestyle behaviours.

Behaviour			Male % (n = 158)	Female % (n = 232)
**Alcohol ever**	**Current**	382	85.2% (132)	86.3% (196)
	**Former**		10.3% (16)	8.4% (19)
	**Never**		4.5% (7)	5.3% (12)
	**Missing n**		3	5
**Alcohol frequency**	**Monthly or less**	357	19.7% (29)	32.9% (69)
	**2–4 times per month**		19.7% (29)	34.8% (73)
	**2–3 times per week**		40.1% (59)	19.5% (41)
	**4+ times per week**		20.4% (30)	12.9% (27)
	**Missing n**		11	22
**Units per day**	**1–2**	341	20.7% (29)	37.3% (75)
	**3–4**		29.3% (41)	23.4% (47)
	**5–6**		11.4% (16)	16.4% (33)
	**7–9**		12.1% (17)	10.0% (20)
	**10+**		26.4% (37)	12.9% (26)
	**Missing n**		18	31
**> recommended units**	**Never**	349	20.5% (30)	31.0% (63)
	**Less than monthly**		28.8% (42)	37.4% (76)
	**Monthly**		19.2% (28)	18.7% (38)
	**Weekly**		26.0% (38)	11.8% (24)
	**Daily or almost daily**		5.5% (8)	1.0% (2)
	**Missing n**		12	29
**Smoking**	**Yes**	385	25.6% (40)	27.5% (63)
	**No**		74.4% (116)	72.5% (166)
	**Missing n**		2	3
**Recreational drugs**	**Yes**	383	38.7% (60)	27.6% (63)
	**No**		61.3% (95)	72.4% (165)
	**Missing n**		3	4
**Cannabis**	**Yes**	389	35.7% (56)	22.0% (51)
	**No**		64.3% (101)	78.0% (181))
	**Missing n**		1	0

**Table 5:** Participant self-report sexual behaviour and health history.

Behaviour			Male % (n = 158)	Female % (n = 232)
**Ever sex**	**Yes**	381	99.3% (152)	98.2% (224)
	**No**		0.7% (1)	1.8% (4)
	**Missing n**		5	4
**Vaginal sex**	**Yes**	367	98.7% (147)	97.7% (213)
	**No**		1.3% (2)	2.3% (5)
	**Missing n**		8	10
**Age first vaginal sex (years)**	**<=15**	336	20.0% (28)	15.8% (31)
	**16–20**		62.9% (88)	73.0% (143)
	**>20**		17.1% (24)	11.2% (22)
	**Missing n**		17	32
**No. vaginal sex partners**	**<=2**	322	16.3% (21)	34.7% (67)
	**3–5**		21.7% (28)	29.0% (56)
	**6–10**		27.9% (36)	19.2% (37)
	**>10**		34.1% (44)	17.1% (33)
	**Missing n**		28	35
**Sexual history**	**Only with females**	375	93.2% (138)	0.0% (0)
	**More often with females**		3.4% (5)	3.5% (8)
	**About equally often with females and males**		0.7% (1)	0.0% (0)
	**More often with males**		2.7% (4)	6.6% (15)
	**Only with males**		0.0% (0)	88.1% (200)
	**Only ever with females and never with males**		0.0% (0)	0.9% (2)
	**I have never had any sexual experience with anyone at all**		0.0% (0)	0.9% (2)
	**Missing n**		10	5
**Oral sex with men**	**Yes**	366	5.3% (8)	89.3% (192)
	**No**		94.7% (143)	10.7% (23)
	**Missing n**		6	13
**Oral sex with men no. of life partners**	**1**	185	0.0% (0)	34.5% (61)
	**2**		12.5% (1)	20.9% (37)
	**3–5**		25.0% (2)	24.9% (44)
	**>5**		62.5% (5)	19.8% (35)
	**Missing n**		0	15
**Oral sex with men no. of partners in past year**	**0**	183	16.7% (1)	36.7% (65)
	**1**		33.3% (2)	58.2% (103)
	**>1**		50.0% (3)	5.1% (9)
	**Missing n**		2	15
**Oral sex with women**	**Yes**	374	90.7% (137)	6.3% (14)
	**No**		9.3% (14)	93.7% (209)
	**Missing n**		6	5
**Oral sex with women no. of life partners**	**<=2**	138	33.1% (41)	71.4% (10)
	**3–5**		32.3% (40)	14.3% (2)
	**>5**		34.7% (43)	14.3% (2)
	**Missing n**		13	0
**Oral sex with women no. of partners in past year**	**0**	140	23.8% (30)	42.9% (6)
	**1**		63.5% (80)	50.0% (7)
	**>1**		12.7% (16)	7.1% (1)
	**Missing n**		11	0
**Anal sex with men**	**Yes**	366	4.7% (7)	24.1% (52)
	**No**		95.3% (143)	75.9% (164)
	**Missing n**		7	12
**Anal sex with women**	**Yes**	149	36.2% (54)	
	**No**		63.8% (95)	
	**Missing n**		8	
**Sexually transmitted infection**	**Yes**	380	10.9% (17)	14.7% (33)
	**No**		89.1% (139)	84.8% (190)
	**Don't remember**		0.0% (0)	0.4% (1)
	**Missing n**		2	8
**Genital warts**	**Yes**	382	7.1% (11)	7.0% (16)
	**No**		92.2% (142)	91.7% (209)
	**Don't remember**		0.6% (1)	1.3% (3)
	**Missing n**		4	4
**HIV Test**	**Yes**	383	20.8% (32)	18.8% (43)
	**No**		76.6% (118)	75.5% (173)
	**Maybe/not sure**		2.6% (4)	5.7% (13)
	**Missing n**		4	3
**HIV Result**	**Negative**	75	96.9% (31)	100.0% (43)
	**Positive**		3.1% (1)	0% (0)
	**Missing n**		0	0
**HPV Vaccine**	**Yes (3 doses)**	231		7.4% (17)
	**Yes (1/2 doses)**			0.9% (2)
	**No**			91.8% (212)
	**Missing n**			1

The self-report vaccine data were comparable to the health administrative datasets. Data linkage of our study data to the Community Health Index (CHI) number (the unique identifier which is a population register used to register all patients in Scotland with a family doctor) was successful on 99.7% (n = 384) of the 385 participants who’s data were submitted to ISD—all of whom had consented for data linkage. For the purposes of preliminary feasibility, linkage to the SIRS database was tested. Of the 375 individuals who completed the questionnaire and linked to SIRS database, 15 had received the HPV vaccination (13 had all three doses, one had two doses, and one had one dose). There were no HPV vaccination records for men in the SIRS data. Among the 221 questionnaires from female participants, 18 reported having had the HPV vaccine (16 reported having had all three doses, 2 had one or two doses), 202 reported no HPV vaccine, with 1 missing value. Three women stated that they had the HPV vaccine (2 with all three doses and 1 with one or two doses) but there is no corresponding record in SIRS; these three women were aged between 35–50 years and it is unlikely that they received the vaccine. Among the 21 women aged 16–25 there was an exact match between the questionnaire and SIRS for 17. Of the remainder, one reported that she had received three doses but has only one record in the SIRS data, and two stated that they had three doses but there is no record in SIRS.

### Follow-up at 6 months

Follow-up is primarily assessed among the 380 participants who had a valid baseline oral gargle/rinse specimen, as the principal aim of the follow-up is in the determination of new/incident acquisitions of oral HPV among participants who were negative at baseline and clearance of HPV among participants who were positive for HPV at baseline. Participants who had a follow-up oral specimen submitted to SHPVRL were considered to have satisfied the conditions for a successful follow-up. Overall follow-up rate was 61.6%, with similar rates among men and women. Follow-up was significantly better among older patients (p<0.0001). There was no evidence (p = 0.186) to suggest that follow-up was associated with deprivation of the participant (Table 6). The evidence suggested that follow-up was better with Research Nurses (p = 0.023) with a rate of 64.3% compared to 48.5% with Dental Care Team model. Follow-up in GDPs was slightly better than outreach teaching centres but not statistically different (p = 0.159). Follow-up by appointment in either setting or by either recruitment model had substantially higher (p<0.0001) follow-up success (86.6%) compared to postal follow-up (39.7%).

**Table 6:** Follow-up participant characteristics.

Variable	Category	Follow-up	HPV Test	%	95% CI	
**Overall**		380	234	61.6	56.6	66.3
**Gender**	**Male**	154	94	61.0	53.2	68.4
	**Female**	226	140	61.9	55.5	68.0
**Age**	**16–25**	39	18	46.2	31.6	61.4
	**26–39**	96	44	45.8	36.2	55.8
	**40–49**	81	49	60.5	49.6	70.4
	**50–59**	83	59	71.1	60.6	79.7
	**60–69**	81	64	79.0	68.9	86.5
**SIMD**	**1 (most deprived)**	89	47	52.8	42.5	62.8
	**2**	57	41	71.9	59.2	81.9
	**3**	55	32	58.2	45.0	70.3
	**4**	89	57	64.0	53.7	73.2
	**5 (least deprived)**	86	55	64.0	53.4	73.3
**Centre**	**Teaching/outreach 1—Tayside**	17	12	70.6	46.9	86.7
	**GDP 1—Tayside**	26	11	42.3	25.5	61.1
	**GDP 2—Tayside**	98	77	78.6	69.5	85.5
	**Teaching/outreach 1—GG&C**	128	72	56.3	47.6	64.5
	**GDP 1—GG&C**	60	36	60.0	47.4	71.4
	**GDP 2—GG&C**	40	21	52.5	37.5	67.1
	**Teaching/outreach 2—Tayside**	11	5	45.5	21.3	72.0
**Recruitment model**	**Research Nurse**	314	202	64.3	58.9	69.4
	**Dental Team**	66	32	48.5	36.8	60.3
**Setting**	**Teaching/outreach**	156	89	57.1	49.2	64.6
	**General Dental Practice (GDP)**	224	145	64.7	58.3	70.7
**Follow Up Method**	**Appointment**	179	155	86.6	80.8	90.8
	**Post**	199	79	39.7	33.2	46.6
	**Unknown**	2	0	0.0	0.0	65.8

### HPV testing process

Generally, operational processes between source collection points and the central testing laboratory were good with few significant issues. Of the samples received at SHPVRL, only 11 could not be processed for HPV testing: 7/11 were discarded due to a packaging error where the transit receptacle was used to contain the sample instead of the primary (inner) tube and 4/11 were labelled inadequately. These issues related to 2 tranches of samples rather than 11 separate errors.

The automated NA extraction procedure, initially designed for cervical cytology samples, was amenable to oral wash pellets and <5% showed clogging/blocking on the automated platform. This level was manageable from a laboratory-process point of view. Quantity of extracted NA varied according to sample and associated cellularity. Quantitative assessment of showed that the mean NA yield was 64ng/ul (range 27ng/ul to 130ng/ul). Yield was not affected according to whether the sample was transported as a postal kit or via the practice and, as described, was more dependent on the individual sample. Purity of the NA as measured by 260/280 absorbance ratios showed between sample variation; however, adequate quality for the key downstream application [HPV PCR testing] was evidenced by amplification of the cellular in-house control (beta globin) in 98% of samples.

### HPV prevalence and diversity

The present study was not powered to examine HPV prevalence of HPV types including in subgroups or in relation to risk associations.There were 380 participants with an unequivocal HPV test result at baseline (Table 7). At baseline, 21 participants were HPV positive, giving a prevalence of 5.5% (95%CI 3.6%, 8.3%). In relation to the HPV types detected at baseline, 17 samples had mono infections, 4 had dual infections and one contained multiple-infection with 5 types. A total of 14 samples contained at least one high-risk HPV type, whereas 5 samples contained low-risk types only (3 x HPV 42, 1 x HPV 44, 1 x HPV 42 & 44) and 2 samples types of intermediate risk (1 x HPV 53, 1 x HPV 66). HPV 16 was the most commonly detected infection and was evident in 12/17 samples; other HR-HPV types detected were HPV 39, HPV 56, HPV 58 and HPV 59 either as mono infections or as part of a multiple infection.

**Table 7:** Oral HPV Prevalence.

Variable	Category	Total	HPV prevalence n	%	95% CI	
**Overall**		380	21	5.5	3.6	8.3
**Gender**	**Male**	154	8	5.2	2.7	9.9
	**Female**	226	13	5.8	3.4	9.6
**Age**	**16–25**	39	1	2.6	0.1	13.2
	**26–39**	96	10	10.4	5.8	18.1
	**40–49**	81	5	6.2	2.7	13.6
	**50–59**	83	1	1.2	0.1	6.5
	**60–80**	81	4	4.9	1.9	12.0
**SIMD**	**1 (most deprived)**	89	9	10.1	5.4	18.1
	**2**	57	1	1.8	0.1	9.3
	**3**	55	5	9.1	3.9	19.6
	**4**	89	4	4.5	1.8	11.0
	**5 (least deprived)**	86	1	1.2	0.1	6.3
	**Missing**	4	1	25.0	1.3	69.9
**HPV Acquisition since baseline**		223	4	1.8	0.7	4.5
**HPV Clearance since baseline**		11	8	72.7	43.4	90.3

Oral HPV positivity did not vary with gender but there is a suggestion of an association with age (p = 0.081) and with deprivation, (p = 0.037). Oral HPV positivity was greatest in the 26–39 age-group where the rate was 10.4% (95%CI 5.8%, 18.1%) compared to a rate of 2.6% (95%CI 0.1%, 13.2%) in the 16–25 age group.

Out of the 380 who gave a valid oral swab at baseline, 234 had a valid swab at follow-up, 61.6% (95% CI 56.6%, 66.3%). Among those with both a baseline and follow up HPV result, 4 out of 223 HPV negative at baseline acquired HPV, 1.8% (95% CI 0.7%, 4.5%). There were 11 HPV positive individuals at baseline and 8 were clear of HPV at follow-up, giving a clearance percentage of 72.7% (95% CI 43.3%, 90.3%).

## Discussion

In summary, our multidisciplinary research group has demonstrated feasibility of recruiting participants using dental care facilities, a centralised laboratory (SHPVRL), and data-linkage resources, to undertake epidemiological studies of oral HPV infection. The experience and data gained now make us well placed to design and implement a population study which would measure the impact of the HPV vaccine on oral HPV among women and assess the implications for extending the vaccine to boys.

We achieved an overall participation rate of 33.1%, which was somewhat lower than the 67% participation rate in the National Health and Nutrition Survey (NHANES) used in the US oral prevalence study [10], but is higher than the approximate 10% reported in the Swedish study [15], and participation rates are unreported in other studies [11, 13, 16]. However, our recruitment strategy was to opportunistically approach sequential patients attending dental practices (for the purposes of dental care), while the NHANES study involved targeted approaches to households and home-based interviews. Lower proportions of younger patients were recruited than anticipated, despite dental registration being high for this group [21]. The 16–25 year age-group was particularly important given that women from this group will have had the opportunity for HPV vaccination as part of the national programme. The imbalance of more female to male patients approached was also interesting. This was similar to data in the British Household Panel Survey 2008 from Scottish responders, which showed that females (71%) were more likely to report having a dental check-up than males (63%), and similarly for NHS dental check-ups: females 54% and males 44% [30]. NHS dental practices in Scotland seem to be a potentially good way to access representative population. In terms of both their location and the profile of registered patients—with limited differences by region, age, sex, and socioeconomic compared to the Scottish population. There is now over 90% of the population registered in 2014, 70% of whom attended in previous 2 years [21].

The consent rate to the data linkage component (96%) was substantially higher than anticipated or observed elsewhere—a systematic review and meta-analysis of the international literature estimated 67% consent rate to use linkage to medical records [31]. Moreover, the data linkage could be used to validate self-reported HPV-vaccination history, but with greater detail on timings and doses available from the linked data than the self-report information.

To our knowledge this was the first observational epidemiological studies in Scotland to recruit patients through dental settings with support of Research Nurses. While both the Research Nurses and Dental Teams faced the same challenges in terms of converting patient approaches to study participants, the Research Nurses were largely able to work more intensively, recruit more participants, and meet their targets on time. Dental Care Teams managed to recruit participants at the same patient participation rate, but were not able to recruit sufficient numbers in the allotted time-frame. The higher costs for the Research Nurse model were due to them being employed and their salary costs being paid directly for this study. They were then deployed to work within the different dental settings. The Dental Care Teams were fitting this study into their clinical daily practice routine and were compensated accordingly on an individual participant recruitment basis which was based on payment of similar tasks on their contract. Completion of the questionnaire on the tablet computer was disappointingly much lower than anticipated. The reasons were that one centre offered the paper version as an option rather than as a back-up, and there were technical reasons, mainly related to internet connectivity issues in two centres, which could be addressed prior to a larger study.

Postal follow-up, in which patients provided their own oral gargle/rinse specimen and posted it (with the online/postal questionnaire) was less effective (36.4%) than a follow-up appointment, However this response was much higher than that observed in an earlier Scottish survey to facilitate HPV vaccine monitoring where response rate to a self-taken urine or vaginal swab was (13.2%) [32]. It is worth noting that recruitment did not seem to be affected by the fact that we were not going to feedback HPV test results.

Participants were able to complete a questionnaire covering a wide-range of factors including demographic, socioeconomic, lifestyle, and sexual behaviour/health. While a third of participants gave feedback that they were uncomfortable with the sexual activity questions, completion rates were high, and participants were able to disclose risk behaviours. Capture of this information may have been facilitated by self-completion in a private area or perhaps (for over a half of participants) by the online questionnaire.

In terms of sexual history, we were able to access a significantly at-risk sample. For example, 39% of male participants reported recreational drug use, 34% of males reported >10 partners, 36% of males reported insertive anal sex with a female partner and 24% of women reported receptive anal sex. The proportions reporting >10 partners is broadly similar to those reported by the NATSAL study [23]. In addition, 15% of women reported a previous STI. This risk behaviour profile carried into reported smoking and alcohol intake, with smoking prevalence (males 26%, females 28%) slightly higher than the Scottish population (males 25%, females 24%), and alcohol abstinence (males 4%, females 5%) lower than the Scottish population (males 12%, females 17%) [24]. There were insufficient data to look at risk associations of sociodemographic and behavioural factors with oral HPV prevalence or incidence.

Our preliminary estimate of oral HPV prevalence (5.5%; 95%CI 3.6, 8.3) is remarkably in-keeping with the overall prevalence in the US population (6.9%; 95%CI 5.7, 8.3) [10], and the pooled estimate from the systematic review and meta-analysis (4.5%; 95%CI 5.7, 4.1) [14]. We did not have sufficient power in our study to produce precise estimates of prevalence in different strata or subgroups, although there was a tendency for higher prevalence in 26–39 years olds (10.4%; 95%CI 5.8%, 18.1).

## Conclusions

Our study demonstrates that it is feasible to investigate the prevalence, incidence, and determinants of oral HPV infection in dental settings. We now know what resources and methods are needed to expand this approach to assess the impact of the school-based HPV vaccination programme in Scotland on oral HPV in girls/women who have received the vaccine and to examine if there has been any herd immunity in boys/men. Crucially we have sufficient data to power a full-population approach. We have provided the first preliminary estimate of oral HPV prevalence in the Scottish population. In our preliminary analysis we found an overall prevalence of oral HPV infection of 5.5% and a higher prevalence among 26–39 year olds of 10.4%.

We demonstrated that the Research Nurse recruitment model was more effective than Dental Care Teams in terms of baseline and follow-up recruitment and meeting recruitment targets on time, but that the former model was more expensive.

To undertake a fully powered study to assess the impact of the HPV vaccination programme in Scotland on oral HPV infection in men and women, we would need to enhance recruitment of 16–25 year olds. This could be established in dental settings serving populations with a high proportion of young people, including community and Further/Higher education settings.

We have also shown that the boundaries of the nature of research undertaken in dental settings can be stretched beyond current traditional dental care and research remits. This points to the importance of further research into barriers and facilitators to implement other risk factor assessment / prevention, screening, and care activities not necessarily associated with current practice (eg: smoking/alcohol counselling, diabetes/HIV screening, or further clinical prevention interventions).

## Supporting Information

S1 File Recruitment and follow-up approach by setting. (DOCX)

S2 File Dental practice payments associated with recruitment models. (DOCX)

S3 File Female Questionnaire. (PDF)

S4 File Male Questionnaire. (PDF)
