# Supplementary material for: Human Papilloma Virus (HPV) Oral Prevalence in Scotland (HOPSCOTCH): A Feasibility Study in Dental Settings
Source: PLoS One. 2016 Nov 18;11(11):e0165847. doi: 10.1371/journal.pone.0165847 (PMC5115665; doi:10.1371/journal.pone.0165847)
Supplement: S1 File — (DOCX) [file pone.0165847.s001.docx]

**SI File 1** Recruitment and follow-up approach by setting

| Dental Setting | Baseline recruitment approach | 6-months follow-up approach |
| --- | --- | --- |
| 2 General Dental Practices  2 General Dental Practices | Research Nurse  Dental Care Team | 2/3 Research Nurse appointment  1/3 Post  2/3 Dental Practice appointment  1/3 Post |
| 3 Dental Outreach / Teaching Centres | Research Nurse | 2/3 Research Nurse appointment  1/3 Post |
