# Supplementary material for: Human Papilloma Virus (HPV) Oral Prevalence in Scotland (HOPSCOTCH): A Feasibility Study in Dental Settings
Source: PLoS One. 2016 Nov 18;11(11):e0165847. doi: 10.1371/journal.pone.0165847 (PMC5115665; doi:10.1371/journal.pone.0165847)
Supplement: S2 File — (DOCX) [file pone.0165847.s002.docx]

**SI File 2** Dental practice payments associated with recruitment models

| Dental Clinical Team Recruitment  Requirement /reimbursement £ | | Research Nurse Recruitment  Requirement /reimbursement £ | |
| --- | --- | --- | --- |
| GCP Training ½ Guild rate  Induction ½ Guild rate | 265  265 | GCP Training ½ Guild rate  Induction ½ Guild rate | 265  265 |
| Payment per patient (Baseline) visit x 65 (target)  Payment per patient (Follow-up) visit X40 (target) [25 postal] | 650 650  400 |  |  |
| Recruitment bonus (Baseline)  Recruitment bonus (Follow-up) | 100  100 |  |  |
| Administration payment | 100 |  |  |
| **Total** | **£1880** | **Total** | **£530** |
