# Supplementary material for: Human Papilloma Virus (HPV) Oral Prevalence in Scotland (HOPSCOTCH): A Feasibility Study in Dental Settings
Source: PLoS One. 2016 Nov 18;11(11):e0165847. doi: 10.1371/journal.pone.0165847 (PMC5115665; doi:10.1371/journal.pone.0165847)
Supplement: S3 File — (PDF) [file pone.0165847.s003.pdf]

HPV Oral Prevalence in Scotland (HOPSCOTCH) Feasibility Study

FEMALE FOLLOW-UP QUESTIONNAIRE

Study ID: ☐☐☐☐☐☐

HPV Oral Prevalence in Scotland (HOPSCOTCH) Feasibility Study

INTRODUCTION

Thank you for taking part in this research study and completing the first questionnaire. It's now time for the second questionnaire: we'd really appreciate you taking the time to complete this too.

Some of the questions in this follow-up questionnaire are similar to those in the first one; however, this questionnaire is shorter. Many questions ask you about what has happened in the last 6 months. Please note that we are looking for your answers to let us know what has happened since you filled in the last questionnaire, so we realise it might be a little shorter or a longer than six months.

Human Papilloma Virus (HPV) is an extremely common virus. Although for many people it may not be harmful, research suggests that in some cases, HPV may be linked to certain types of cancer. In our research, we are particularly interested in HPV that is found in the mouth and throat. We need to know more about how many people in Scotland are carrying the HPV virus and how it is passed from person to person.

In the future, we plan to ask a large number of dental patients across Scotland to take part in a full scale study that will help us to answer these questions. Before we can do this, we need to make sure that our research plan will work. This kind of study has not been conducted with dental patients before, so we need to be sure that we can get enough people to take part and that we can collect the information that we need.

You can skip any question you do not want to answer. When you have finished, please seal your questionnaire in the envelope provided and return it to the research nurse / dental health worker. They will post it using secure postal services to the University of Glasgow and will never see your answers.

You can find more information about the study on the Patient Information Sheet. If you need any help please let the research nurse/ member of the dental team know.

To begin...

**1. Are you male or female?**	☐ Male<br>☐ Female<br>***Note: this is the questionnaire for females***

The following are questions about your oral health (your mouth and throat). Your answers will help us to understand if oral health is linked to oral HPV.

**2. In the last 6 months have you started wearing a denture?**	☐ Yes...***go to Q2(a)***<br>☐ No....***go to Q3***
**2(a) In the upper jaw do you wear...**	☐ Full denture<br>☐ Partial denture<br>☐ No denture
**2(b) In the lower jaw do you wear...**	☐ Full denture<br>☐ Partial denture<br>☐ No denture
**3. How often do you clean your teeth?**	☐ Never<br>☐ Less than once a week<br>☐ 1 to 2 times a week<br>☐ Every other day<br>☐ Once a day<br>☐ 2 times a day<br>☐ 3 times a day<br>☐ More than 3 times a day
**4. Do your gums bleed when you clean your teeth?**	☐ No<br>☐ Sometimes<br>☐ Always or almost always
**5. In the last 6 months have you had heartburn?**<br>*That is, a burning pain or discomfort felt behind your breastbone that may move up into your throat.*	☐ Yes...***go to Q5(a)***<br>☐ No....***go to Q6***
**5(a) If Yes, how frequently?**	☐ At least once a day<br>☐ 2 to 6 times per week<br>☐ Once per week<br>☐ Less than once per week
**6. In the last 6 months have you suffered from regurgitation?**<br>*That is, a sour or bitter-tasting acid backing up into your throat or mouth, also known as acid reflux.*	☐ Yes...***go to Q6(a)***<br>☐ No....***go to Q7***

**6(a) If Yes, how frequently?**	☐ At least once a day<br>☐ 2 to 6 times per week<br>☐ Once per week<br>☐ Less than once per week
**7. How often do you use mouthwash?**	☐ Never<br>☐ Less than once a week<br>☐ 1 to 2 times a week<br>☐ Every other day<br>☐ Once a day<br>☐ 2 times a day<br>☐ 3 times a day<br>☐ More than 3 times a day

The next section asks questions about alcohol, smoking and recreational drugs. Your answers will help us to understand if any of these are linked to oral HPV.

Alcohol questions:

**8. Have you ever drunk alcohol? How would you describe yourself?**	☐ I currently drink (including occasional drinking) or have stopped drinking within the last 12 months***...go to Q9***<br>☐ I used to drink (including occasional drinking) but stopped over 12 months ago***...go to Q9***<br>☐ I have never drunk alcohol***...go to Q12***

For those who have stopped drinking alcohol, questions 9, 10 and 11 refer to the period before you stopped drinking alcohol.

**9. How often do you (did you) have a drink containing alcohol?**	☐ Monthly or less	☐ 2 - 4 times per month	☐ 2 - 3 times per week	☐ 4+ times per week

This is 1 unit of alcohol…

1 unit

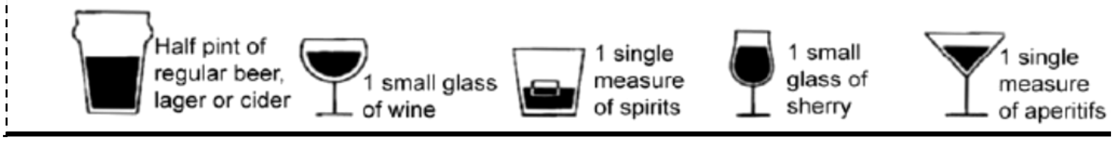

…and each of these is more than 1 unit

more than 1 unit

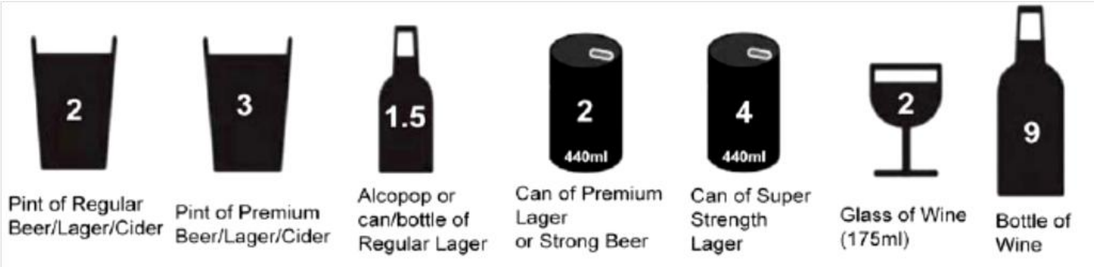

**10. In the last year, how many units of alcohol did you drink on a typical day when you were drinking?**	☐ 1-2	☐ 3–4	☐ 5–6	☐ 7–9	☐ 10+
**11. How often have you had 6 or more units on a single occasion in the last year?**	☐ Never	☐ Less than monthly	☐ Monthly	☐ Weekly	☐ Daily or almost daily

Smoking questions:

**12. In the last 6 months have you smoked cigarettes?** *Please include roll-ups but exclude cigars.*	☐ Yes...***go to Q12(a)*** ☐ No....***go to Q13***
**12(a) About how many cigarettes a day do you usually smoke?**	☐☐☐ ***If you smoke more at the weekend, put down the weekend number.***
**13. In the last 6 months have you used any other tobacco products?**	☐ Yes...***go to Q13(a)*** ☐ No….***go to Q14***
**13(a) If yes, which ones? *(tick all that apply)***	☐ Pipe tobacco ☐ Cigars ☐ Chewing tobacco ☐ Other, please write in: ________

Recreational drugs questions:	
**14. In the last 6 month have you taken recreational drugs?** *There is no need to tell us about drugs you have been given on prescription.*	☐Yes...***go to Q14(a)***<br>☐No....***go to Q15***
**14(a) If yes, which ones?** *Tick all that apply.*	☐**Cannabis** (marijuana, pot, grass, skunk, dope, hash, blow, spliff, weed, joints)<br>☐**Stimulant** drugs (e.g. cocaine, crack, crystal meth, ecstasy, amphetamines, or poppers)<br>☐**Psychedelic** substances (e.g. LSD, magic mushrooms or Ketamine)<br>☐**Downers or tranquilisers** (e.g. Temazepam or Valium)<br>☐**Opiates** (e.g. Heroin or Methadone)

The next section asks about your sexual health. Your answers will help us to understand if oral HPV is related to sexual health.	
**15. In the last 6 months have you been told by a doctor or other healthcare professional that you had a Sexually Transmitted Infection (STI)?**	☐Yes<br>☐No
**16. In the last 6 months have you been told by a doctor or other healthcare professional that you had Genital Warts (venereal warts)?**	☐Yes<br>☐No
**17. In the last 6 months have you had a test for HIV (the virus that causes AIDS)?** *This does not include testing when giving blood.*	☐Yes...***go to Q17(a)***<br>☐No....***go to Q18***<br>☐Maybe/not sure...***go to Q18***
**17(a) If yes, what was the result of that test?**	☐Positive<br>☐Negative<br>☐Don't know
**18. In the last 6 months have you been vaccinated against cervical cancer (received HPV vaccine)?**	☐Yes - I have completed three doses of the vaccine<br>☐Yes - I have had one or two doses of the vaccine but not all three doses<br>☐No

The next set of questions is about kissing and sexual activity. Your answers will help us to understand more about how sexual behaviours are linked to oral HPV.

Please answer as many questions as you feel able.

**19. I have had some sexual experience...**<br>*Choose one statement that is true for you.*<br>*Include any experience that you felt was sexual, such as kissing, touching, intercourse or any other form of sex.*	☐ Only with males, never with females...***go to Q20***<br>☐ More often with males, and at least once with a female... ***go to Q20***<br>☐ About equally often with males and females***... go to Q20***<br>☐ More often with females, and at least once with a male... ***go to Q20***<br>☐ Only ever with females and never with males... ***go to Q20***<br>☐ I have never had any sexual experience with anyone at all... ***go to Q26 (page 7)***
**20. In the last 6 months have you kissed someone with an open mouth?**	☐ Yes...***go to Q20(a)***<br>☐ No....***go to Q21***
**20(a) How many people have you kissed with an open mouth in the last 6 months?**	☐

For questions 21-25 please only tell us about any sex (that is vaginal intercourse, oral sex or anal sex) that happened in the last 6 months. Please include everyone you have had sex with, whether it was just once, a few times, a regular partner, your civil partner or husband.

**21. In the last 6 months have you had sex with either a man or woman?**<br>*That is vaginal intercourse, oral sex or anal sex.*	☐ Yes...***go to Q22***<br>☐ No....***go to Q26 (page 7)***
**22. In the last 6 months have you had oral sex with a man?**<br>*That is, putting your mouth on a man's genital area (penis).*	☐ Yes...***go to Q22(a)***<br>☐ No....***go to Q23***
**22(a) In the last 6 months with how many men have you had oral sex?**	☐
**22(b) When having oral sex, in the last 6 months, did you use a condom?**	☐ Never/Rarely<br>☐ Always/Usually
**23. In the last 6 months have you had oral sex with a woman?**<br>*That is, putting your mouth on a woman's genital area.*	☐ Yes...***go to Q23(a)***<br>☐ No....***go to Q24***
**23(a) In the last 6 months with how many women have you had oral sex?**	☐
**23(b) When having oral sex, in the last 6 months, did you use protection (i.e. a latex square or dam)?**	☐ Never/Rarely<br>☐ Always/Usually

**24. In the last 6 months have you had vaginal sex?** *That is, a man's penis in a woman's vagina.*	☐Yes...***go to Q24(a)*** ☐No....***go to Q25***
**24(a) In the last 6 months with how many men have you had vaginal sex?**	☐
**24(b) When having vaginal sex, in the last 6 months, did you use a condom?**	☐Never/Rarely ☐Always/Usually
**25. In the last 6 months have you had anal sex with a man?** *That is, a man's penis in a female partner's anus (rectum or back passage).*	☐Yes...***go to Q25(a)*** ☐No....***go to Q26***
**25(a) In the last 6 months with how many men have you had anal sex?**	☐
**25(b) When having anal sex, in the last 6 months, did you use a condom?**	☐Never/Rarely ☐Always/Usually

The next section asks a few questions about you.

**26. What is your legal marital or same-sex civil partnership status?**	☐ Never married and never registered a same-sex civil partnership<br>☐ Married<br>☐ Separated but still legally married<br>☐ Divorced<br>☐ Widowed<br>☐ In a registered same-sex civil partnership<br>☐ Separated but still legally in a same-sex civil partnership<br>☐ Formerly in a same-sex civil partnership which is now dissolved<br>☐ Surviving partner from a same-sex civil partnership
**27. What is the highest educational level you obtained?**	☐Primary School<br>☐Secondary School<br>☐Further Education/Technical College<br>☐University<br>☐Some other type of college, please write in: ☐
**28. Are you currently working**	☐Yes<br>☐No

29. What was the occupation/job which you had for the longest time in your life?

Please write in:

30. From all sources, what is your total annual household income?

- ☐ Nil or loss
- ☐ £1 to £9,999
- ☐ £10,000 to £19,999
- ☐ £20,000 to £29,999
- ☐ £30,000 to £39,000
- ☐ £40,000 or more

31. What proportion of your household income (including your own) would you say comes from benefits?

- ☐ None
- ☐ Very Little
- ☐ About a quarter
- ☐ About half
- ☐ About three quarters
- ☐ All

32. Please write in your postcode.

☐☐☐☐ ☐☐☐☐

This will be used to work out the how much poverty there is in the area in which you live. It will not be used to look up your address or to identify you in any way.

33. Any final comments?

The end! Thank you very much for taking the time to complete this questionnaire.

Please seal your questionnaire in the envelope provided and return it to the research nurse / dental health worker. They will never see your answers and will post it securely to the research team.
